# Management of severe Raynaud's Phenomenon secondary to autoimmune vasculopathy in a young woman

**DOI:** 10.4314/gmj.v55i1.16

**Published:** 2021-03

**Authors:** Maame-Boatemaa Amissah-Arthur, Lily P Wu

**Affiliations:** 1 School of Medicine and Dentistry, University of Ghana Medical School, Accra, Ghana

**Keywords:** Acute limb ischaemia, autoimmune, connective tissue disease, secondary Raynaud's phenomenon, digital vasculopathy

## Abstract

Raynaud's phenomenon as a cause of acute limb ischaemia in the warmer climates of Sub-Saharan Africa region is uncommon because it is usually thought of as a disease common in cold weather. The prevalence of connective tissue diseases among Black Africans is increasing, and these conditions are associated with secondary Raynaud's phenomenon and ischaemic digital lesions. We present the case of a 36-year old female with dermatomyositis/systemic sclerosis overlap and secondary Raynaud's phenomenon who presented with acute limb ischemia (wet gangrene of all digits) in a Tertiary Hospital in Ghana. Young patients presenting with acute limb ischaemia should also be screened for an underlying connective tissue disease. In patients with connective tissue disease, the onset of digital vasculopathy can be rapid and progressive, hence treatment must be prompt and comprehensive to enable better clinical outcomes.

**Funding:**

None declared

## Introduction

Connective tissue diseases (CTD) are a secondary cause of Raynaud's phenomenon and a significant cause of acute limb ischaemia (ALI) through mechanisms of underlying vasculopathy and resultant permanent tissue damage. Many of these disorders, like systemic sclerosis, mixed connective tissue disease, systemic lupus erythematosus, and dermatomyositis, are presented more commonly in clinical practice.^1^–^3^ Coupled with a warmer climate, Raynaud's phenomenon, which is largely a cold weather disease, has been infrequently reported in the Sub-Saharan African region. The outcome of ALI depends on the clinical stage and the underlying CTD, with some conditions linked to poorer prognosis than others.^4^,^5^ In patients with connective tissue disease, the onset of digital vasculopathy can be rapid and progressive. This report focuses on severe digital ischaemia in a Ghanaian patient with dermatomyositis/systemic sclerosis overlap managed in a tertiary hospital.

## Case Report

A 36-year old female secretary presented with a ten month history of rash on the face, muscle weakness and arthralgia. Examination revealed hyperpigmentation with bilateral periorbital swelling, indicative of an heliotrope rash. There was symmetrical proximal muscle weakness-grade 4/5, mild skin tightening affecting the dorsum of the hands, puffiness of the fingers and abnormal nailfolds. Clinical features suggested dermatomyositis/systemic sclerosis overlap, confirmed by raised antinuclear antibody (1:320) and myositis-specific antibody (anti-PL12- 58.0 U/ml and anti-Ro52– 75.0 U/ml) titres. In addition, ESR was elevated (101 mm/hr) as were the muscle enzymes (CK - 686 IU/L; LDH 668 IU/L). The full blood count, renal, liver and lipid profile tests were within normal limits. (Table 1) Hydroxychloroquine 400mg daily, azathioprine 75mg daily, prednisolone 20mg daily, omeprazole 20mg daily and a calcium supplement were commenced. Four months later, she presented acutely with one-week history of pain in all digits and paraesthesia and numbness, which worsened in cold temperature. She had no risk factors for atherosclerosis. There was no history of recurrent arterial/venous thromboembolism or adverse pregnancy outcomes suggestive of anti-phospholipid syndrome (APS). She was not on any medication that predisposed her to vasoconstriction in the digits.

**Table 1:** Summary of Key Laboratory Investigations

LABORATORY INVESTIGATIONS					
**FULL BLOOD COUNT**	HB 10.1g/dL (10–14)	WBC 10.5 X 10^9^ /L (4–10)	PLT 353 x 10^9^/L (150–400)		
**MUSCLE-SPECIFIC ENZYMES**	CK 686 IU/L (<170)	LDH 668 IU/L (135–214)	AST 91 IU/L (0–32)		
**MYOSITIS-SPEICIFIC AUTOANTIBODY** **TESTS**	ANTI-PL12 (58.0 U/ml) STRONGLY POSITIVE	ANTI-RO 52 (75.0 U/ml) STRONGLY POSITIVE	ANTI-MI2 NEGATIVE	ANTI-JO1 NEGATIVE	
**AUTOANTIBODY** **TESTS**	ANA 1:320	ANTI-dsDNA NEGATIVE	ANTI-SM NEGATIVE	ANTI-CCP 2.8 U/ml (<7.0)	RHEUMATOID FACTOR POSITIVE
**AUTOANTIBODY** **TESTS**	ANTI-RO/SS-A NEGATIVE	ANTI-LA/SS-B NEGATIVE	ANTI-U1RNP NEGATIVE	ANTI-Scl 70 NEGATIVE	CENTROMERE AB NEGATIVE
**INFLAMMATORY MARKERS**	CRP 120.9 mg/L (<5)	ESR 101 mm/hr (0–20)			
**RENAL** **FUNCTION**	SODIUM 142 mmol/L (135–145)	POTASSIUM 4 mmol/L (3.5–5.1)	UREA 2.9mmol/L (2.1–7.1)	CREATININE 57.1ummol/L (44–80)	URINE ACR 1.7mg/mmol (< 2.8)
**OTHER INVESTIGATIONS**	HBSag NEGATIVE	HCV NEGATIVE	HIV 1&2 NEGATIVE	LIPID PROFILE NORMAL	

**Figure 1:**
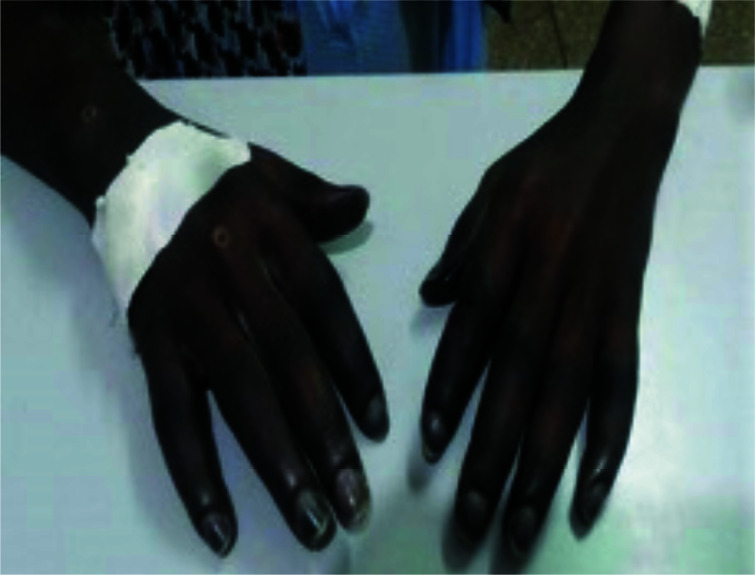
Dusky insensate fingers

On examination, there was cyanosis and gangrene of the fingers, nail fold infarcts and digital ulcers in the pulp of the fingers. Figure 1 All ten toes were gangrenous. symmetrical in both arms (average 104/70 mmHg); pulse rate was 112 beats/min and regular with no radio-radial delay.

Blood pressure was normal and There was full complement of peripheral pulses in both lower limbs, with no stigmata of chronic ischaemia. Venous thromboembolism was excluded on doppler ultrasound scan. An angiogram showed patent lower limb arteries with complete foot arches bilaterally, but no patent digital arteries on both sides. A diagnosis of acute limb ischaemia due to secondary Raynaud's phenomenon (Rutherford III disease) with an acute flare of dermatomyositis/systemic sclerosis was made.

**Figures 2 and 3:**
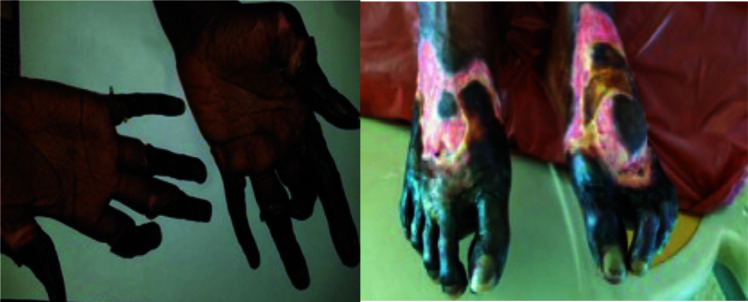
Gangrene of all fingers and toes

Nifedipine 20mg bd, fluoxetine 20mg od and topical glyceryl trinitrate paste were prescribed to alleviate the symptoms. Prednisolone dose was increased from 20mg to 40mg daily to treat the underlying CTD. Despite this treatment, her condition deteriorated rapidly over days to a few weeks. Bosentan 62.5mg bd, naftiduryl oxalate 200mg tds and Aspirin 75mg daily were added to her treatment. Azathioprine was discontinued and treatment was escalated to intravenous methylprednisolone followed by cyclophosphamide. Subsequent pulses of cyclophosphamide were discontinued due to severe wound infection despite an intensive, daily wound dressing schedule and antibiotic therapy (Figures 2 & 3).

**Figure 4:**
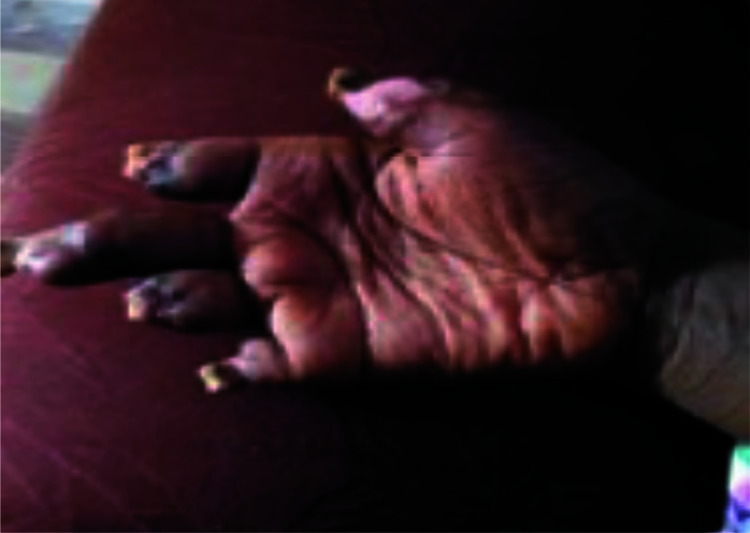
Autoamputation of all 5 fingers of the right hand

**Figure 5:**
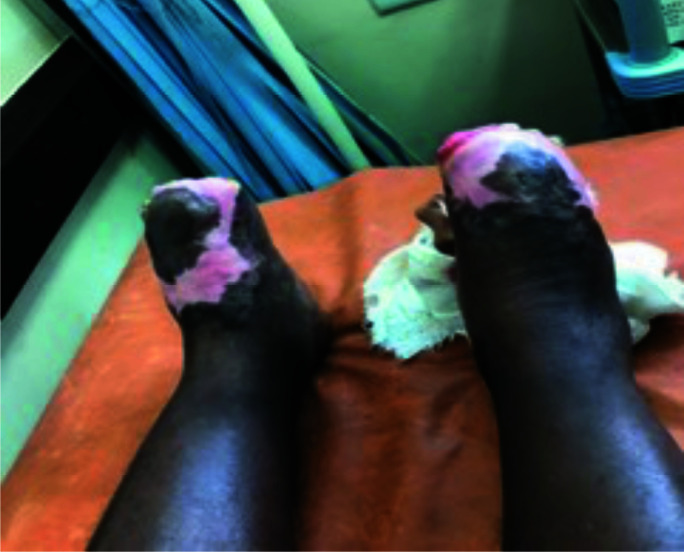
Healed ischemic ulcers and autoamputation of all ten toes

The surgical plan was to optimally manage the tissue necrosis to allow wound healing and auto-amputation of the digits rather than pursue below knee amputations. Healing eventually occurred, after several weeks, with autoamputation occurring at various levels (Figures 4 and 5). She was discharged after 4 months with reviews at the Rheumatology, Vascular and Plastic Surgery outpatient departments. She was re-admitted a year later for refashioning of the digits to remove exposed phalanges.

## Discussion

Raynaud's phenomenon (RP) is a severe vaso-occlusive disorder of digital arteries due to an interplay of multiple mechanisms and is triggered by cold, smoking and emotional stress.^6^ Secondary RP associated with connective tissue diseases, is complicated by obliterative vasculopathy, intravascular microthrombi and autoimmune vasculitis which can lead to irreversible ischaemia and digit loss.

Secondary RP is a prominent clinical criterion in the diagnosis of some of the CTDs like systemic sclerosis and MCTD and occurs in over 90% of such cases.^7^ There are predictors of vasculopathy that should alert the clinician and inform early therapy, particularly in the absence of classic signs. These are high autoantibody titres- antinuclear antibody (ANA); anticentromere, anti-topoisomerase, anti-Th/To, anti-RNA polymerase III antibodies; anti-RNP antibody; antinuclear cytoplasmic antibody (ANCA); and anti-beta2-glycoprotein 1.^8^ In addition, the presence of avascular areas on nailfold capillaroscopy caused by capillary loss and digital infarcts indicate underlying digital ischaemia.^8^

Digital ulcers (DU), pitting scars and pulp loss are reported in 30–50% of patients with systemic sclerosis^9^ and in 15–20% of the patients with recurrent DUs, the digital ulcers will progress to irreversible tissue loss and gangrene or amputation of one or more digits.^10^ Critical digital ischaemia occurs in about 1.6% of patients with CTDs. In this case, the patient did not report the triphasic colour change, pain, numbness or paraesthesia associated with RP at the start of her illness, however nailfold abnormalities were detected, indicative of underlying vasculopathy. It is important to recognise these clinical signs early and advise the patient on lifestyle changes. They should be told to avoid cold exposure, as the small changes in ambient temperature can lead to significant vasospasm^11^; avoid smoking which is linked to the onset and severity of RP and DUs^12^; and avoid other triggers such as caffeine, beta-blockers and sympathomimetic drugs.

Beyond conservative management, vasodilators should be used early to stall the progression of ischaemia. Oral dihydropyridine calcium channel blockers (CCB) significantly reduce the frequency and severity of RP attacks and are usually the first-line agents to use.^13^ Fluoxetine (selective serotonin reuptake inhibitor) is helpful as an adjunct to calcium channel blockers and can be used when low BP restricts optimal dosing of Nifedipine as was observed in the case.^14^

For this patient, severe Raynaud's phenomenon with critical ischaemia of the digits developed rapidly and recommended therapy at this stage of presentation is Iloprost, a prostacyclin analogue, due to the quick effect it has on digital arterial dilatation and inhibition of platelet aggregation.^11^,^15^ It prevents or reduces the formation of new DUs (25% vs. 33% placebo).^16^ Iloprost is not readily available in Ghana and all attempts to get hold of it at the time proved futile. During this time, Bosentan, an endothelin receptor antagonist, was added due to its effect on reducing new ulcer formation.^10^,^17^ For severe refractory ischaemia as in this case, Sildenafil, a phosphodiesterase 5 inhibitor, is normally added.^18^ Its effect is also to improve perfusion leading to complete resolution of DUs and lower the risk of new ulcer formation.^19^,^20^ Combination therapy of Bosentan and Sildenafil was not possible for her on account of persistently low blood pressure.

Other agents used to minimize vasoconstriction include the 5-HT2 receptor blocker (Naftiduryl oxalate) and phosphodiesterase-3 inhibitor (Cilostazol). Generally, these drugs are used as additive treatment in situations where first-line vasodilator therapy is ineffective or unavailable as was in our case. Adjunctive drugs such as statin therapy can be used if there is evidence of atherosclerosis^21^; anti-platelet therapy due to its inhibitory effect on platelet aggregation in cases where tissue viability is threatened; however warfarin has not been proven for the use in limb ischaemia due to digital vasculopathy caused by underlying CTD and in the absence of an arterial or venous thrombus, it was not indicated in the patient's management. Finally, locally applied topical glyceryl trinitrate significantly improves digital blood flow through its direct effect on nitric oxide causing symptomatic relief for the patient.^22^

Alongside managing the aggressive vasculopathy, the underlying CTD should be treated adequately, and in her case, the oral dose of prednisolone was increased and subsequently switched to intravenous methylprednisolone. Intravenous cyclophosphamide was introduced and these measures were used to curb the inflammatory drive to the vascular component of the disease. Secondly, acute digital ischaemia is often complicated by secondary wound infections hence, antibiotics should be considered early to aid healing. Her recovery was fraught with setbacks due to the extensive wound infections she developed which limited the used of cyclophosphamide after the first cycle.

The overall management of this case required continuous vascular and plastic surgical inputs from the onset to inform the surgical care plan. Digital sympathectomy^23^ and botulinum toxin injection^24^ could not be pursued. The decision was made to observe closely as cessation of ongoing ischaemia had commenced; allow resolution of the infected areas; and complete auto-amputation, rather than intervene prematurely with bilateral below knee amputations, as often, the critically ischaemic digit may autoamputate. Eventual tissue loss was less than initially expected allowing for limb and function preservation. This enabled her to remain ambulant and independent with her activities of daily living.

## Conclusion

Raynaud's phenomenon may be difficult to detect in dark skin tones. In the clinical setting, the presence of nailfold infarcts and digital ulcers should raise suspicion for early identification of underlying digital vasculopathy. Though therapeutic options may be limited due to availability or cost, early intervention is key to halt the rapidly progressive limb ischaemia. A multidisciplinary approach to the management of vasculopathy in the presence of a threatened digit and the underlying CTD is necessary for a successful outcome for the patient.
